# Academy of Natural Sciences

**Published:** 1841-11-06

**Authors:** 


### ACADEMY OF NATURAL SCIENCES.

The Academy of Natural Sciences of Philadelphia, an institution perhaps the most vigorous, and certainly the most liberally accessible of its class in the United States, publishes a monthly bulletin of its proceedings, containing earlier notices of some of its more important memoirs than can be derived from its more voluminous "Transactions," and giving a sketch of lighter labours and incidental remarks, not deemed appropriate for the periodical volumes, though possessing, in many instances, no ephemeral interest. This bulletin, designed chiefly for the use of its members and other professional naturalists, often contains matter of high importance to medicine or the collateral sciences; and it is our duty, as journalists, to make the medical public acquainted with such portions of the proceedings of the Academy as are peculiarly fitted for our columns. No apology need be offered for antedating the moment, by commencing with extracts from the early numbers of the bulletin. They will apologise for themselves. When, in the succeeding year, we commence giving an account of the scientific institutions and charities which should be considered as tributary to the medical school of Philadelphia, the Academy will be canvassed among the number.

*Ordinary Meeting, June* 1, 1841.

Dr. Morton made some remarks on the Ancient Peruvians; that extraordinary people who preceded the Inca race, and whose monuments show a remarkable advance in civilization at a very early epoch.

"In my work on American skulls (Crania Americana,) I have expressed the opinion that the heads of the ancient Peruvians were *naturally* very much elongated; and that they differed in this respect from those of the Inca Peruvians, and other surrounding nations; and having given this opinion at a meeting of the Academy prior to the publication of my work, I take this occasion to renounce it.

In the American Journal of Science, for March, 1840, I have already, in a brief note, adverted to this change of opinion; and I now repeat my matured conclusions in connection with positive facts derived from the work of a distinguished traveller and naturalist, M. Alcide D'Orbigny.

This gentleman not only visited the elevated table-land of the Andes, which was once inhabited by the ancient Peruvians, but he remained a long time in that interesting region, and has collected numerous facts in relation to the people themselves.

1. The descendants of the ancient Peruvians yet inhabit the land of their ancestors, and bear the name of Aymaras, which was probably their primitive designation.

2. The modern Aymaras resemble the surrounding Quichua or Peruvian nations, in colour, figure, features, expression, shape of the head, (which they have ceased to mould into artificial forms,) and in fact in every thing that relates to physical conformation and social customs; their languages differ, but even here there is a resemblance which proves a common origin.

3. On examining the tombs of the ancient Aymaras, in the environs of the lake Titicaca, M. D'Orbigny remarked that those which contained the compressed and elongated skulls, contained also a greater number that were not flattened; whence he infers that the deformity was not natural, or characteristic of the nation, but the result of mechanical compression.

4. It was also remarked that those skulls which were flattened were uniformly those of men, while the heads of the women always retained the natural shape,—the squared or spheroidal form which is characteristic of the American race, and especially of the Peruvians.

5. The most elongated heads were found in the largest and finest tombs; showing that the deformity was a mark of distinction among these people.

6. The researches of M. D'Orbigny confirm the statements made at distant intervals of time by Pedro de Cieza, Garcilaso de la Vega, and Mr. Pentland, and prove conclusively, what I have never doubted, that these people were the architects of their own tombs and temples; and not, as some suppose, intruders who had usurped the civilization and appropriated the ingenuity of an antecedent and more intellectual race.

M. D'Orbigny found temples from 100 to 200 metres in length, facing the east, and ornamented with rows of angular columns; enormous gateways made of a single mass of rock, and covered with bas reliefs; colossal statues of basalt; and large square tombs, wholly above ground, and in such numbers that they are compared to towns and villages.

My published observations go to show that the internal capacity of the cranium, as indicative of the size of the brain, is nearly the same in the ancient and modern Peruvians, viz. about seventy-five cubic inches, a smallness of size which is without a parallel among existing nations, excepting only the Hindoos.

M. D'Orbigny even supposes the ancient Peruvians to have been the lineal progenitors of the Inca family; a question which is not yet decided. Supposing this to be the fact, we may inquire how it happens that the Incas should have so entirely abandoned the practice of distorting the cranium; especially as this, among the Aymaras, was an aristocratic privilege?

I was at first at a loss to imagine how this singular elongation of the head was effected; for when pressure is applied to a spheroidal head, as in the instance of the Chenouks and other tribes of the Columbia river, the skull expands *laterally* in proportion as it is depressed above; whereas, in those people, the head is narrow from the face to the occiput. It seems probable that this conformation was produced by placing splints or compresses on each side of the head from the cheek bones to the parietal protuberances, and another on the forehead, and confining them by rotary bandages. In this way the face, in the process of growth, would be protruded in front, and the head elongated backwards; while the skull, in all other directions, could expand comparatively little.

Dr. Goddard suggested that the deformity observable in this series of crania, might have been produced by the action of rotary bandages alone, without the use of splints or compresses. Dr. Morton admitted the possibility of this result in some of the heads, but thought that in others there was satisfactory evidence of the use of the splint or compress, especially on the os frontis.

Dr. Morton exhibited, in further illustration, six casts of heads and three skulls of these people, all of which present the elongated form in question. For further details, Dr. Morton referred to his Crania Americana, and to the beautiful and instructive work of M. D'Orbigny, entitled, "L'Homme Americain, considéré sous ses rapports physiologiques et moraux." These works were at the same time placed on the table for inspection and comparison.

*Stated Meeting, June 8.*

Dr. Blanding mentioned some facts in relation to the surprising fecundity of the striped Bass, Labrax lineatus. He obtained one of these fish which weighed 65½ lbs. and the roes 4 lb. 6 oz. 2 dr. He found one hundred of the eggs to weigh a grain and a half; and although one end of the containing membrane had been ruptured, whereby some hundreds were lost, the whole number of eggs must have been at least 2,248,000.

*Stated Meeting, July 6, 1841.*

*Verbal Communications.*—Dr. Morton (Prof. Johnson taking the chair) made the following communication.

"I submit to the inspection of the members eight skulls of the ancient Mexican race, for six of which I am indebted to Don J. Gomez de la Cortina, and for the other two to Dr. John P. Macartney of the city of Mexico. All these crania have been received since the publication of my Crania Americana.

The skulls are of the following nations.

1. *Otomies.*—Four in number, with the high vertex, flat occiput, great lateral diameter and broad faces, characteristic of the American race. The Otomies preceded the Tolticas, and were the least cultivated of the demi-civilized nations of Anahuac. The largest of these heads gives 92 cubic inches of internal capacity; the smallest, that of a female, only 67.

2. *Chechemecan.*—A single skull, of 83 cubic inches of internal capacity. This nation followed the Tolticas in the possession of Mexico in the 11th century of our era. They were nomades and hunters, but rapidly acquired the arts and civilization of their predecessors.

3. *Tlascalan.*—A single cranium. These people formed one of the seven tribes who established themselves in Mexico during the Chechemecan monarchy, and are now renowned in history for their warlike exploits. They are well known to have rendered Cortez essential aid in taking the city of Mexico. This skull gives an internal capacity of 84 cubic inches, and, like the others of this series, is remarkable for its diameter between the parietal bones.

It is worthy of remark that the average internal capacity of these six authentic Mexican skulls, is precisely what I have accorded to these people in my Crania Americana, viz., seventy-nine cubic inches. The mean of the facial angle also accords with my previous measurements, and gives 75°.

All these heads were obtained from tumuli or mounds, within the territories of the nations whose names they bear, so as to leave no doubt in the mind of the distinguished gentleman from whom I received them, of their having pertained to individuals of those nations.

The two remaining crania are supposed to be those of Aztecks, who also belonged to the confederacy of the seven tribes, but were the last to take possession. These were the people who subsequently obtained the supreme power, and under the name of Aztecks or Mexicans, governed the country at the epoch of the Spanish invasion, A. D. 1521. The Aztecks were a brave and intelligent people, but remarkable for bloody rites, both in their warlike and religious observances. They were less cultivated than the Tolticas, but much more so than the surrounding barbarous tribes; and appear, in fact, to have been the connecting link between the two. The largest of these heads gives 85 cubic inches of internal capacity; the smallest 77; the medium being 80 cubic inches. The configuration of these heads is on the same model as the preceding series, and the mean facial angle differs but a single degree.

Whoever will be at the pains to compare this series of skulls with those from the barbarous tribes, will, I think, agree that the fact thus derived from organic characters, corroborate the position I have long maintained, that all the American nations, excepting the polar tribes, are of one race and one species, but of two great families, which resemble each other in physical but differ in intellectual character."

*Stated Meeting, August 17, 1841.*

Dr. Morton (Dr. Coates taking the chair) made some remarks on the sutures of the cranium as connected with the growth of the corresponding bones.

He adverted to the opinion long in vogue, that the chief use of the sutures was to facilitate the process of parturition : a theory which is refuted by the fact that they exist in the skulls of all the oviparous animals as strongly marked as in those of the viviparous class. That they give a certain increased facility to parturition is unquestionably true; but their more uniform function is to subserve the growth of bones, which they do by osseous deposition at their margins; hence a suture in the cranium is equivalent to the surface which intervenes between the shaft and epiphysis of a long bone. The latter grows in length by deposition at its extremities, and the epiphysis disappears, like the suture of the cranium, when the growth of the bone is completed.

Dr. Morton illustrated these views by means of the skull of a mulatto boy who died when about eighteen years of age. In this instance the sagittal suture is entirely wanting; in consequence of which the lateral growth of the cranium has ceased in early infancy, (no doubt when the suture became consolidated,) so that the diameter between the parietal protuberances is less than 4.5 inches, instead of 5, which is about the negro average. The squamous sutures, however, are fully open, whence the skull has continued to expand in the upward direction until it has reached the full vertical diameter of the negro, viz., 5.5 inches. The coronal suture is also wanting, excepting some traces at its lower or lateral extremities. The result of this deficiency is seen in the very inadequate development of the forehead, which is low and narrow, but elongated below by means of the various craniofacial sutures. The lambdoidal suture is complete, thus permitting of posterior elongation; and the growth in this direction, together with the great vertical diameter already mentioned, has allowed the brain to attain the bulk of seventy-seven cubic inches, or six or eight inches short of the negro average.

The growth of the brain and that of the skull are of course concentaneous; nor is it probable that either could be developed without the sutures; hence there is reason to believe that the absence of these may be a cause of idiocy, by preventing the growth of the brain, and thus impairing or destroying its functions.

Dr. Coates (B. H.) inclined to the opinion that, in cases similar to those presented by Dr. Morton, the disappearance of the sutures was rather to be regarded as a consequence than a cause; and took place, as in old age, because the necessity for further extension of growth no longer existed, from the final cessation of enlargement in the brain. Uniting with Dr. Morton in the belief that the office of the sutures was to permit a more rapid development and growth of the cranium, by allowing ossification to go on from several centres at the same times, the bones of the skull, in this respect, resembling the trunk and epiphyses of a long bone, Dr. Coates inclined, at the same time, to the double belief that growth and other changes took place, not at the sutures only, but throughout the whole extent of a living cranial bone. The parietal bone of a newly-born infant was not mathematically of the same shape with the central portion of that of an adult. Were the brain, in one of the cases referred to by Dr. Morton, to acquire, by any means, a further enlargement, it ought to be presumed, in the present state of our physiological knowledge, that the bone would enlarge to a corresponding extent; and it would be, therefore, inferred that the ossification of the sutures would not limit the growth of the brain.

This view Dr. Coates endeavoured to illustrate by a comparison with the opinion of Mr. Serres, that the relative and successive growth of the parts of the brain were a *consequence* of the relative size of their arteries during the period of formation; in regard to which, he believed, physiologists were much agreed in the conclusion that the development of a portion of the brain and of its corresponding artery were concentaneous processes; but that if any priority in causation were to be allowed, it should be assigned to the organ; in consequence of the existence and comparative size in outline of which, it became necessary if the ability of the system permitted, that a proportionate supply of blood should be furnished to the part, through a vessel of a suitable size, in order to afford new materials for enlargement. Primary growth, he imagined, took place in the interstitial substance, and that the larger arterial branches, and even the capillaries were rather an instrument or adjuvant than a cause. The formation of additions to existing solids would thus resemble that of the primordia of the fœtus, near which no vessels of the parent are observed, while the vascular appearances are found to approach the newly organized individual at a period subsequent to its formation.

Dr. Coates called the attention of the Academy to the whiteness, thinness, and semi-transparency of the specimen exhibited by Dr. Morton, in all the lines usually exhibiting the sutures. This he considered, not only as indicating the previous existence of real sutures, but as corresponding with the views entertained, by some late comparative anatomists, in regard to the analogy of parts. He alluded to those who believe the analogous parts in animal formations to exist to a very great extent indeed, although composed of very diversified materials, and adapted to very different purposes in the various beings in which they exist.

